# Pathomechanisms of SARS-CoV-2 infection and development of atherosclerosis in patients with COVID-19: A review

**DOI:** 10.1097/MD.0000000000031540

**Published:** 2022-12-09

**Authors:** Alicja Zofia Gospodarczyk, Celina Wojciechowska, Kamil Piotr Marczewski, Natalia Justyna Gospodarczyk, Jolanta Zalejska-Fiolka

**Affiliations:** a Department of Biochemistry, Faculty of Medical Sciences in Zabrze, Medical University of Silesia, Zabrze, Poland; b Second Department of Cardiology, Faculty of Medical Sciences in Zabrze, Medical University of Silesia, Zabrze, Poland; c Department of Emergency Medicine, Faculty of Medical Sciences in Katowice, Medical University of Silesia, Katowice, Poland.

**Keywords:** atherosclerosis, cardiovascular system, COVID-19, SARS-CoV-2

## Abstract

The pandemic of coronavirus disease 2019 (COVID-19) has posed a major health challenge for over 2 years. The severe acute respiratory syndrome coronavirus 2 (SARS-CoV-2) that causes it belongs to single-stranded ribonucleic acid viruses and causes acute respiratory distress syndrome. The initial outbreak was discovered in December 2019 in Wuhan province, where SARS-CoV-2 quickly spread to other countries. In addition to respiratory disorders, it has been shown that during and after COVID-19 infection, cardiovascular diseases are often developed or exacerbated, such as: arterial hypertension, coronary artery disease, arrhythmias, heart failure and thromboembolic complications. In view of the higher prevalence of atherosclerosis in patients with COVID-19, we described the pathomechanisms of the development of this infection and the possible correlations between SARS-CoV-2 infection and thromboembolic complications. We focused on the role of the inflammatory response, renin-angiotensin system and endothelial dysfunction in the development of atherosclerosis in patients with COVID-19. A thorough understanding of the hemodynamic mechanisms and the impact of the infection on the cardiovascular system will allow for the proper selection of appropriate therapy in patients after SARS-CoV-2 infection.

## 1. Introduction

Infectious diseases have been accompanied by humans for centuries. In 2002 to 2003, an epidemic of severe acute respiratory syndrome (SARS) caused by SARS-CoV emerged in China.^[1]^ The current challenge has become a pandemic caused by a new coronavirus, severe acute respiratory syndrome coronavirus 2 (SARS-CoV-2), whose primary outbreak of infection was discovered in Wuhan Province, People’s Republic of China in December 2019. The disease that is caused by the infection is called the coronavirus disease 2019 (COVID-19).^[2,3]^ As of late 2019. SARS-CoV-2 has rapidly spread to many countries, causing a worldwide pandemic that currently poses a threat to global public health owing to high human mortality rates and near-total shutdown of economic and social activities.^[4]^ According to the World Health Organization (WHO), as of today (May 16, 2022), SARS-CoV-2 has become the cause of more than 521 million confirmed cases of infection and 6,2 millions deaths worldwide, of which in Poland the number of cases is 6,003,436 and there are more than 116 thousand deaths.^[5]^

Coronaviruses are single-stranded ribonucleic acid viruses that infect both humans and animals. There are 4 subfamilies, namely alpha-, beta-, gamma-, and delta-coronaviruses, of which the alpha- and beta-groups are specifically derived from bats, while the gamma- and delta-coronaviruses are derived from pigs and birds. Of all the subtypes that cause human infections, alpha-coronaviruses cause asymptomatic or mild infection, while the beta-group causes severe illness and death. SARS-CoV-2 belongs to the beta coronavirus group.^[2,3,6]^ COVID-19 is a clinical syndrome that initially presents with fever, cough, nasal mucosal congestion, fatigue, and other symptoms of upper respiratory tract infections. In approximately 81% of patients, the infection may progress to a severe form with dyspnea and chest auscultatory changes consistent with pneumonia.^[1]^ In addition, loss of smell and taste and gastrointestinal disorders, such as vomiting, diarrhea, and abdominal pain are frequently reported in 2% to 10% of patients, with diarrhea and nausea preceding the development of fever and respiratory symptoms in 10% of patients.^[7]^ Confirmation of viral pneumonia may be provided by tests such as decreased blood oxygen saturation (reduced saturation), abnormalities in arterial blood gasometry, and changes seen on chest radiography.^[2]^ In addition to pneumonia, patients may develop severe symptoms of acute respiratory distress syndrome (ARDS) which is the leading cause of mortality in patients with COVID-19. More specifically, the clinical spectrum is very broad and ranges from the complete absence of symptoms to severe symptoms of pneumonia and ARDS, leading to multi-organ failure and sepsis requiring treatment in the intensive care unit.^[2,8]^

To date, there are several studies analyzing the course, mechanisms of infection, and complications. However, there are few studies that have clarified the association between COVID-19, atherosclerosis and acute coronary syndrome.^[9]^ This review provides an overview of the current knowledge on the possible pathophysiological links between SARS-CoV-2 infection and atherosclerosis development. This topic is of great importance because of its potential to reduce the mortality due to COVID-19.

### 1.1. Covid-19 and cardiovascular diseases

Yang et al showed that the most common comorbidities with COVID-19 were hypertension (21.1%), diabetes (9.7%), cardiovascular disease (8.4%), and respiratory disease (1.5%).^[10]^ Moreover, many studies have emphasized that patients with severe SARS-CoV-2 infection are at a higher risk of cardiovascular disease. Wang et al demonstrated that myocardial damage alone, defined as elevated cardiac troponins, new electrocardiography or echocardiographic changes, was found in 7.2% of the individuals.^[7]^ In many studies, the authors agreed that age and comorbidities are associated with both a severe disease course and a higher risk of death in patients infected with SARS-CoV-2.^[9]^ SARS-CoV-2 infection undoubtedly affects cardiovascular functions. The possible cardiac symptoms in patients with COVID-19 include: acute coronary syndrome (ST segment elevation myocardial infarction or no ST elevation acute coronary syndrome); arrhythmias; heart failure; cardiogenic shock; pericardial effusion with or without tamponade; and thromboembolic complications.^[11]^ In the article published in June 2020 Schiavone et al described possible pathophysiological links between SARS-CoV-2 infection and acute coronary syndromes (ACS). They emphasized the role of increased oxygen demand, inflammation, and the influence of other respiratory infections on the pathogenesis of ACS. This study emphasizes the importance of inflammation not only in the development of ACS, but also in atherosclerosis.^[9,12]^ Therefore, research should focus on evaluating the pathomechanisms contributing to the development of COVID-19 and identifying possible correlations between SARS-CoV-2 infection and atherosclerosis.

### 1.2. Patogenesis 1 – immunopathogenesis

The immune response to SARS-CoV-2 infection appears to play a key role in the pathogenesis of the disease. After SARS-CoV-2 entry, a series of antiviral immune responses are activated, initiated by the innate immune system, which induces the production of pro-inflammatory cytokines in response to foreign pathogens. The immune system consists of T lymphocytes, which can directly kill virus-infected cells, and B lymphocytes, which produce pathogen-specific antibodies. During the immune response, among the cytokines produced, we distinguish chemokines, which further attract inflammatory cells to sites of infection to induce an inflammatory response. Although these phenomena are fundamental in removing viruses from the body, they can also lead to damage to normal host tissues.^[4,13]^

Several scientific papers have reported that individuals with severe COVID-19 develop the following immunological changes: lymphocyte activation and dysfunction, granulocyte and monocyte abnormalities, and increased cytokine and immunoglobulin production.^[4,14,15]^ As early as 2004. Li et al demonstrated that a reduction in peripheral T-lymphocyte subsets is characteristic of patients with severe SARS infection, whereas cured patients have rapid recovery of this population.^[16]^ Similarly, in the case of SARS-CoV-2 infection, comparing lymphocyte levels showed that compared to mild and asymptomatic cases, lymphopenia was more frequently observed in patients with a severe course, especially a reduced number of cluster of differentation 4 T, cluster of differentiation 8 T, natural killer cells and B lymphocytes.^[17]^ In contrast, a study by Reynard et al found that Ebola patients had significantly elevated levels of pro-inflammatory cytokines, while recovered patients showed low levels.^[18]^ As a result, immune response factors are now recognized as potential biomarkers of COVID-19 progression.^[16,18]^

Several potential mechanisms are responsible for lymphocyte dysfunction. It has been demonstrated that SARS-CoV-2 infects human respiratory epithelial cells through interactions between its surface spike glycoprotein (S protein) and the angiotensin-converting enzyme 2 (ACE2) receptor located on epithelial alveolar cells.^[19]^ In addition, SARS-CoV-2 can directly enter the T lymphocytes and macrophages. Therefore, it has been hypothesized that the expression of the ACE2 receptor on T lymphocytes promotes the entry of SARS-CoV-2 into lymphocytes.

In another study, the number of T lymphocytes was shown to be inversely related to the levels of interleukin 6 (IL-6), interleukin 10 (IL-10), and tumor necrosis factor α (TNF-α), indicating that increased levels of pro-inflammatory cytokines may promote the depletion of T lymphocyte populations.^[20]^ Another cause that may inhibit lymphocyte proliferation is the elevated lactic acid levels found in the blood of patients with severe COVID-19.^[13,18]^ A study by Fischer et al examined the effect of lactic acid on the function of cytotoxic T lymphocytes. Activated T lymphocytes were shown to metabolize glucose mainly by glycolysis, leading to intracellular production of lactic acid. Export of lactic acid outside the cell occurs via the monocarboxylate transporter-1 (MCT-1), which depends on the concentration gradient. It has been observed that when the concentration of external lactic acid is high, MCT-1 is blocked and intracellular lactic acid accumulation occurs, thereby disrupting T-cell metabolism and function.^[21]^

Another study also showed significantly higher levels of neutrophils in patients with a complicated disease course, while the percentages of eosinophils, basophils and monocytes were reduced. This fact can be explained by the association with lymphopenia, which can lead to microbial infection, further activating and inducing neutrophil recruitment in patients’ blood.^[22,23]^ These data suggest that lymphocyte dysfunction is associated with worse prognosis in patients with COVID-19. By examining cytokine levels, it has been shown that the most severe cases of COVID-19 are characterized by extremely high levels of pro-inflammatory cytokines, such as the interleukins: interleukin 1 β (IL-1β), interleukin 2, IL-6, interleukin 7, interleukin 8, IL-10, granulocyte colony-stimulating factor, granulocyte-macrophage colony-stimulating factor, IP10 protein, monocyte chemotactic protein 1, macrophage inflammatory protein-1α, interferon-γ and TNF-α, representing a “cytokine storm.” In particular, high concentrations of IL-1β, IL-6 and IL-10 have been reported.^[24–27]^ IL-6 is a key cytokine that stimulates acute-phase proteins-producing in the liver, such as C-reactive protein and fibrinogen, causing increased blood coagulation, which is responsible for the high probability of acute deep vein thrombosis and pulmonary embolism in patients with COVID-19.^[28]^

The diagnosis of COVID-19 is based on the detection of SARS-CoV-2 nucleic acids in nasopharyngeal swabs, and specific antibody titers (immunoglobulin M and immunoglobulin G) to SARS-CoV-2 can be measured in the blood. In a study by Zhang et al, higher immunoglobulin G titers were found to correlate strongly with disease severity, thus providing a simple marker to classify patients into severe and non-severe cases. In another study, increased total antibody levels were independently associated with a worse clinical course in COVID-19 patients.^[23,29]^ In conclusion, as a result of immune abnormalities that develop in response to SARS-CoV-2 infection, such as lymphopenia, lymphocyte dysfunction, and granulocyte and monocyte abnormalities, microbial infections, septic shock, and severe multi-organ dysfunction can occur.^[4,30]^ Therefore, it is important to understand the mechanisms underlying immune abnormalities in COVID-19 patients so that treatments can be instituted to manage the immune response to SARS-CoV-2 and ultimately enhance antiviral immunity while suppressing systemic inflammation.

### 1.3. Patogenesis 2 – renin-angiotensin system

ACE2 is a functional SARS-CoV-2 receptor that allows viral entry into the human cells. It is present in many tissues, but its highest expression has been observed in the lung epithelial cells, blood vessels, small intestinal enterocytes, macrophages, monocytes and lymphocytes.^[31]^ The rapid spread of SARS-CoV-2 can be explained by the high viral titer during the viremia phase, leading to massive invasion of human alveolar epithelial cells via an ACE2 protein-binding mechanism. Consequently, during the acute phase of the disease, the virus leads to deterioration of respiratory function, causing pneumonia.^[8]^ The severity of infection ranges from mild symptoms, such as cough and dyspnea, to ARDS, a manifestation of critical illness. The ubiquity of the ACE2 enzyme and patient susceptibility can lead to multiorgan failure, including acute myocardial infarction and/or myocarditis and liver and kidney damage, resulting in systemic disorders.^[32]^ The increased risk of COVID-19 in obese individuals is also explained by the wide distribution of ACE2 in small intestinal enterocytes.^[33]^ The immune system becomes over-activated during the acute phase of infection, leading to the production of significant amounts of cytokines and chemokines. Due to the high affinity for the ACE2 receptor during massive viral replication, the postulated pathogenetic mechanisms are directed at the alveolar epithelial cells, endothelium, lymphocytes and macrophages.^[31]^ Virus fusion with ACE2 results in a decrease in its expression, as it becomes involved in SARS-CoV-2 entry into cells, leading to dysfunction of the renin-angiotensin system and increased vascular permeability. Under normal conditions, ACE2 is involved in the modulation of the renin-angiotensin-aldosterone system, which is responsible for the conversion of angiotensin II to angiotensin (1–7), resulting in vasodilatory, anti-inflammatory, antithrombotic, antiproliferative, antifibrotic, and antiarrhythmic effects.^[34]^ ACE2 deficiency is associated with an increase in the concentration of angiotensin II, which is not utilized in the enzymatic reaction, causing opposing effects and leading to adverse effects, such as hypertension, increased vascular permeability, hypertrophy, fibrosis, and an increased inflammatory response. Thus, reduced ACE2 expression may also contribute to the progression of atherosclerosis and cardiovascular disease. These findings may be supported by the documented deficiency of ACE2 in the African population, which explains the high prevalence of severe symptoms and high mortality among this community with COVID-19.^[31,33,35]^ In addition, ACE2 is involved in the inactivation of bradykinin as a component of the kallikrein-kinin system, causing pulmonary edema due to a local increase in vessel wall permeability, which increases the risk of developing ARDS, sepsis and multi-organ failure.^[36]^

### 1.4. Role of endothelial dysfunction

The vascular endothelium is a layer of cells that lines the inner wall of blood vessels and acts as a barrier between blood and tissues. Under physiological conditions, it performs many important functions in the body, such as maintaining cardiovascular homeostasis by regulating the transport of cells, nutrients and metabolites, and the secretion of biologically active substances. In addition, the endothelium plays a key role in maintaining hemostatic balance by influencing the coagulation and fibrinolysis processes, preventing blood loss during vascular injury, and inhibiting excessive thrombosis through multiple anticoagulant pathways, including the protein C and S pathways.^[37]^ Furthermore, endothelial cells (EC) interact with platelets and leukocytes, causing their activation and adhesion to the site of vascular injury caused by injury, inflammation or infection. Cardiovascular risk factors such as dyslipidemia, obesity, diabetes, hypertension and smoking lead to endothelial dysfunction. Its dysfunction causes several abnormalities, including increased vascular permeability, pro-thrombotic effects, metabolic changes, and induction of cytokines and adhesion molecules with the uptake of inflammatory cells from the circulation.^[38,39]^ Endothelial cells play an important role in regulating vascular tone by synthesizing and releasing vasodilatory substances such as nitric oxide (NO) and prostaglandins, as well as vasoconstrictors such as endothelin and angiotensin II.^[40]^ The vascular endothelium is a major source of circulating nitric oxide NO, which is formed by endothelial nitric oxide synthase-3. In states of increased oxidative stress, the formation and stability of endothelial nitric oxide synthase-3 are impaired, leading to a decrease in the concentration of NO, a factor with vasoconstrictive properties. Thus, the excessive formation of reactive oxygen species relative to antioxidants is considered a hallmark of EC dysfunction. Under these conditions, endothelial protective properties are impaired with concomitant pro-inflammatory, pro-thrombotic and atherosclerotic activation in the blood vessels, which is directly implicated in the pathogenesis and progression of cardiovascular disease.^[38,41]^ Recent evidence also suggests that EC dysfunction is a central pathophysiological process in many viral infections, including COVID-19. SARS-CoV-2 infection causes endothelial dysfunction at multiple levels, including inflammatory activation, cytokine storms, leukocyte infiltration, increased vascular permeability, platelet aggregation, thrombosis, vasoconstriction, reactive oxygen species production and apoptosis.^[37]^ An argument for the involvement of ECs in the course of COVID-19 is their role in the inflammatory process, which is also a key factor of the so-called “cytokine storm” in ARDS and many cardiovascular pathologies. In addition, the pro-thrombotic activities and disseminated intravascular coagulation observed in COVID-19 patients reflect endothelial dysfunction, in which junctions are loosened and EC cohesion is lost. This, in turn, leads to the exposure of subepithelial pro-thrombin materials, adhesion factors responsible for platelet adhesion and activation, and thrombin, which stimulates the coagulation cascade.^[42]^ The COVID-19 “cytokine storm” results in direct exposure of the endothelium to cytokines, causing inflammation and vascular damage. This process can lead to EC death, which contributes to increased vascular permeability, driving the endothelial inflammatory response, resulting in a source of cytokines such as IL-1 and IL-6, which are characteristic of the cytokine storm in SARS-CoV-2 infection. Thus, we provide further evidence of the involvement of EC in the pathogenesis of COVID-19.^[43]^

It seems that it would be beneficial to develop an anti-inflammatory therapy, as confirmed by the RECOVERY trial showing therapeutic success with low-dose dexamethasone in patients as 1^st^-line treatment in the UK.^[44]^ Endothelial dysfunction of the vessel wall contributes to the loss of antithrombotic properties. In particular, a decrease in the secretion of anti-aggregative prostacyclins and a proaggregative increase in thromboxane synthesis by activated platelets may favor the predominance of pro-thrombotic and pro-inflammatory processes. Furthermore, it has been shown that under certain inflammatory conditions, ECs express their surface adhesion receptors, such as von Willebrand factor, contributing to the activation and adhesion of thrombocytes to the intact endothelium. This process may lead to secondary recruitment of circulating leukocytes by platelets, forming complexes that impede vascular blood flow. It has been hypothesized that in small blood vessels, these ternary aggregates (EC thrombocytes-leukocytes) are sufficient to cause inadequate microvascular perfusion in the lungs and other organs. Reduced myocardial perfusion is revealed by elevated troponin levels, which are frequently observed in COVID-19 patients and are associated with a worse course and prognosis.^[45,46]^ Moreover, intravascular thrombosis may further damage the endothelium, confirming the role of ECs in SARS-CoV-2 infection, as some patients show severe endothelial dysfunction associated with a procoagulant state. Thus, it seems appropriate to introduce antithrombotic or platelet aggregation inhibition therapy in COVID-19 patients to improve endothelial function and therapy focusing on suppressing EC inflammation, resulting in a reduced pro-thrombotic effect in SARS-CoV-2 infection.^[37,47]^

### 1.5. Atherosclerosis

Many studies have confirmed increased mortality due to COVID-19 in patients with atherosclerosis. However, few studies have addressed the correlation between SARS-CoV-2 infection and the development of thromboembolic events. Atherosclerosis is an inflammatory process characterized by lipid accumulation within the arterial walls. Modifiable risk factors include hyperlipidemia, obesity, hypertension, diabetes, physical inactivity, and cigarette smoking.^[48]^ In the first stage of atherosclerosis development, as a result of chronic endothelial damage by risk factors, there is increased permeability of the vessel walls to low-density lipoproteins and leukocytes (monocytes and lymphocytes), which accumulate in the subendothelial space. The lipoproteins are then oxidized, and blood monocytes migrate to the inner membrane, where they are transformed into macrophages and foam cells. The next step is calcification of these foci, leading to local hardening of the arteries and the formation of atherosclerotic plaques that are prone to rupture. Above the atherosclerotic focus, endothelial defects appear, which become areas of adherence and aggregation of platelets, promoting thrombosis. This process leads to narrowing of the arterial lumen and obstruction of blood flow, which can cause acute coronary syndrome or stroke.^[49,50]^ Atherosclerosis is a major disease common in patients with COVID-19, and several studies have confirmed that SARS-CoV-2 infection increases mortality in patients with atherosclerosis.^[8]^ It has also been reported that COVID-19 can cause myocardial infarction/inflammation, heart failure, and arrhythmias in patients with atherosclerosis.^[51]^ A study conducted in Wuhan by Chen et al showed that heart failure was observed in approximately 4% of patients, whereas acute myocardial damage was revealed in 7% to 20% of patients with COVID-19. In turn, myocardial damage puts patients at a risk of developing atherosclerosis.^[52]^ The most commonly reported complication was hypertension, which was observed in approximately 40% of the patients.^[7]^ The effect of SARS-CoV-2 on lipid metabolism and atherosclerosis development was demonstrated in an experiment investigating the long-term effects of infection by Wu et al Based on a study evaluating patients 10 years after SARS-CoV infection in 2003, increased free fatty acids and phosphatidylglycerols were observed in patients due to altered lipid metabolism.^[53,54]^ In addition, increased levels of cardiac injury biomarkers, such as troponin, are found, and some patients are diagnosed with myocardial infarction and acute myocarditis based on clinical examination, cardiac MRI, and echocardiographic changes.^[55,56]^ SARS-CoV-2 triggers an inflammatory response in COVID-19 patients, enhancing platelet activation and increasing susceptibility to the development of atherosclerosis associated with chronic inflammation of the arterial wall.^[57]^ Worldwide reports suggest that susceptibility to COVID-19 is increased in patients with preexisting cardiovascular disorders. Endothelial dysfunction is a common link between a predisposition to atherosclerosis; therefore, preexisting dysregulation may be an independent risk factor for the severity of SARS-CoV-2 infection. The link between these conditions may be due to the fact that atherosclerosis is a chronic inflammatory disease of the endothelium, characterized by infiltration, deposition of deposits and lipid oxidation, stimulating a self-perpetuating inflammatory state.^[8,58]^ In atherosclerosis, the inflammatory signaling pathways toll-like receptor 4/nuclear factor kappa *β* and JAK/STAT are activated, which exacerbates inflammation, leading to increased cytokine production and subsequent activation of immune cells. Consequently, the immune system is disrupted due to chronic inflammation, potentially increasing susceptibility to COVID-19.^[59]^ In patients with atherosclerosis, secreted pro-inflammatory IL-1*β* induces the expression of downstream cytokines such as IL-6, TNF-*α*, interleukin 8 and chemokines, which lead to increased lipid deposition in the vessel wall and macrophage cholesterol overload, consequently exacerbating local inflammation and plaque instability.^[60]^ Moreover, atherosclerosis-activated monocytes produce additional amounts of IL-1*β*, IL-6, and TNF-*α*, whose increased levels correlate with COVID-19 progression. These immune processes may play a key role in increasing host susceptibility to cytokine storm development and worsening of COVID-19 progression. Although further studies are needed, the above hypothesis may partially explain the severity of SARS-CoV-2 infection in patients with atherosclerosis.^[8,61]^

## 2. Summary

According to the WHO, cardiovascular disease is the leading cause of death worldwide. Among these, the highest mortality rate is characterized by ischemic heart disease, 98% of which is caused by coronary artery atherosclerosis.^[62]^ Existing or newly diagnosed atherosclerosis places an additional burden on COVID-19 patients.

Currently, COVID-19 is 1 of the most serious social and economic problems. Although the pandemic has been ongoing for more than 2 years, the relationship between SARS-CoV-2 infection and atherosclerosis remains unclear. Therefore, it is important to deepen our knowledge and search for the possible causes of the severe course of COVID-19. In this paper, we describe the mechanisms and processes that with high probability explain the correlation between the course of COVID-19 and the occurrence of complications in the form of thromboembolic incidents. The situation regarding SARS-CoV-2 infection is changing dynamically, and although new publications appear every day, improving our knowledge of the course of COVID-19 may turn out that the data available at the time of writing will be outdated in the future. Further research is needed, and researchers should focus on highlighting the role of inflammation in the development of COVID-19 and atherosclerosis to effectively suppress the excessive inflammatory response and prevent future disease complications. A thorough understanding of the hemodynamic mechanisms and the effects of infection on the cardiovascular system will allow for the proper selection of appropriate therapy in patients after COVID-19.

## Author contributions

**Conceptualization:** Alicja Zofia Gospodarczyk, Celina Wojciechowska, Kamil Piotr Marczewski, Natalia Justyna.

**Data curation:** Kamil Piotr Marczewski, Natalia Justyna, Jolanta Zalejska-Fiolka.

**Formal analysis:** Kamil Piotr Marczewski, Natalia Justyna, Jolanta Zalejska-Fiolka.

**Project administration:** Alicja Zofia Gospodarczyk, Natalia Justyna, Jolanta Zalejska-Fiolka, Jolanta Zalejska-Fiolka.

**Supervision:** Celina Wojciechowska, Jolanta Zalejska-Fiolka.

**Validation:** Celina Wojciechowska, Kamil Piotr Marczewski.

**Visualization:** Alicja Zofia Gospodarczyk, Celina Wojciechowska, Kamil Piotr Marczewski, Natalia Justyna.

**Writing – original draft:** Alicja Zofia Gospodarczyk, Kamil Piotr Marczewski, Natalia Justyna, Jolanta Zalejska-Fiolka.

**Writing – review & editing:** Alicja Zofia Gospodarczyk, Celina Wojciechowska, Kamil Piotr Marczewski, Natalia Justyna, Jolanta Zalejska-Fiolka.
